# Characteristics and influencing factors of sleep disturbance in breast cancer patients: a cross-sectional study

**DOI:** 10.3389/fonc.2026.1749557

**Published:** 2026-03-24

**Authors:** Yi Xie, Yan Wang, Yuhang Fang, Guanghui Zhu, Bailu Sui, Xinhe Yuan, Yu Chen, Mengyang Li, Wei Fan, Ying Zhang

**Affiliations:** Guang’anmen Hospital, China Academy of Chinese Medical Sciences, Beijing, China

**Keywords:** breast cancer, characteristics, cross-sectional study, influencing factors, sleep disturbance

## Abstract

**Background:**

With prolonged survival of breast cancer patients, symptom management is increasingly pivotal. Sleep disturbance, a prevalent and persistent quality-of-life-impairing symptom, remains incompletely elucidated, thus this study aims to clarify its clinical characteristics and influencing factors.

**Methods:**

A total of 150 breast cancer patients were recruited. Sleep disturbance was assessed using the Pittsburgh Sleep Quality Index (PSQI). Pain was evaluated with the Visual Analogue Scale (VAS), fatigue via the Revised Piper Fatigue Scale (PFS-R), and emotional status through the Hospital Anxiety and Depression Scale (HADS). Multivariate logistic regression was conducted to ascertain associated factors.

**Results:**

Approximately 68.67% of patients exhibited “moderate” or “severe” sleep disturbance (PSQI score >10), with difficulty falling asleep as the predominant type of sleep disturbance. Univariate analysis showed that receipt of ovarian function suppression (OFS) and/or aromatase inhibitor (AI) therapy, pain, fatigue, anxiety, and depression were associated with exacerbated sleep disturbance, while multivariate analysis revealed that each 1-point increase in pain, fatigue, and depression scores was independently associated with a 23.7%, 25.0%, and 23.7% higher risk of severe sleep disturbance, respectively.

**Conclusion:**

Sleep disturbance is highly prevalent and severe in breast cancer patients, with difficulty falling asleep as the core feature. Clinical practice should focus on patients with the aforementioned influencing factors, prioritizing interventions targeting shortened sleep latency.

## 1 Introduction

According to the 2022 global cancer epidemiological data, breast cancer ranks first in terms of incidence among female cancers, accounting for 11.6% of all new cancer diagnoses worldwide with approximately 2.3 million new cases annually (1), posing a severe threat to women’s physical and mental health. Owing to advances in early screening and comprehensive treatment modalities, the 5-year overall survival rate of breast cancer patients in developed countries has exceeded 85%, while data from developing countries also continues to rise (2, 3). With the prolongation of survival time, boosting quality of life and reducing symptom burden have become core priorities for both patients and healthcare providers, among which the management of common persistent symptoms is particularly crucial (4).

Sleep disturbance is among the most prevalent and long-lasting symptoms among breast cancer patients, with approximately 50%-70% experiencing varying degrees of sleep disturbance throughout the entire disease trajectory (including at diagnosis, during active therapy, and in the long-term follow-up phase) (5, 6). Compared with the general population, sleep disturbance in this population is characterized by greater complexity and refractoriness (7). A 12-month longitudinal study revealed that after the initial curative treatment for breast cancer, most patients’ psychological indicators such as depression, distress, and anxiety improved over time, yet they still suffered from sleep disturbances (8). Without timely and effective intervention, sleep disturbance not only exacerbates other symptoms and reduces quality of life but also may impair immune function (9, 10), affect treatment adherence (11), and significantly elevate the likelihood of cancer recurrence (12). Therefore, early identification, assessment, and targeted intervention of sleep disturbance are crucial for enhancing patient outcomes and quality of life.

Previous studies have identified several potential influencing factors for sleep disturbance in breast cancer patients, including demographic factors (13), treatment-related factors (14), and symptom-related factors (15). Specifically, demographic factors such as age, economic status and lifestyle factors have been reported to be closely associated with the occurrence and severity of sleep disturbance in breast cancer patients (13). However, the existing evidence still has obvious limitations: first, most studies are confined to specific treatment phases or specific populations, such as patients with lymphedema (16), surgical patients (17), or chemotherapy patients (18), whereas sleep disturbance affects the entire disease trajectory, not just during specific treatment periods. Second, the correlations between sleep disturbance and other symptoms remain insufficiently explored. Breast cancer patients often present with a co-occurring “sleep disturbance-pain-fatigue-emotion” symptom cluster (19, 20), but most existing studies only focus on the correlation between emotions and sleep disturbance. Finally, the majority of studies merely report the prevalence of sleep disturbance (21) without in-depth analysis of its clinical characteristics (such as the severity of sleep disturbance and specific symptom types), resulting in limited guiding value for clinical interventions. Therefore, there is a pressing need exists to carry out research covering multidimensional factors and diverse samples to provide more robust evidence for the accurate identification and prevention of sleep disturbance in breast cancer patients.

This study hypothesizes that sleep disturbance is highly prevalent and severe in breast cancer patients, with demographic, disease-related, treatment-related and symptomatic factors contributing to its aggravation. The primary objective is to systematically characterize the clinical features of sleep disturbance in breast cancer patients. The secondary objectives are to identify its potential influencing factors via univariate and multivariate analyses, and clarify the independent associated factors for severe sleep disturbance. This work is intended to provide empirical evidence and clinical insights for the targeted prevention, management and intervention of sleep disturbance in this population.

## 2 Methods

### 2.1 Study design

Conducted in line with the Helsinki Declaration and STROBE Statement (Vandenbroucke et al., 2007) (22), this cross-sectional study secured ethical clearance from the Ethics Committee of Guang’anmen Hospital, China Academy of Chinese Medical Sciences (No. 2024-027-KY). Study participants were recruited and evaluated from the Oncology Clinic of Guang’anmen Hospital, China Academy of Chinese Medical Sciences, between March 2024 and October 2025.

### 2.2 Participants

Inclusion criteria: ① Histopathologically confirmed breast cancer (3); ② Diagnosed with sleep disturbance (23) with a total score of ≥6 on the Pittsburgh Sleep Quality Index (PSQI); ③ Female; ④ Aged ≥18 years; ⑤ Karnofsky Performance Status (KPS) score ≥60; ⑥ Able to cooperate with scale completion; ⑦ Volunteered to participate and submitted signed written informed consent.

Exclusion criteria: ① Pre-existing sleep disturbance prior to breast cancer diagnosis; ② Complicated with brain metastasis; ③ Concomitant infectious or autoimmune diseases; ④ Comorbid with severe primary diseases such as heart, brain, liver, and kidney diseases.

This study only enrolled breast cancer patients with sleep disturbance (PSQI ≥ 6), and the subsequent analysis focuses on the severity distribution of sleep disturbance in this symptomatic population rather than the prevalence of sleep disturbance in the overall breast cancer population.

### 2.3 Measures

#### 2.3.1 General information

The collected data included age, height, weight, KPS score, and degree of treatment cost burden.

#### 2.3.2 Breast cancer information

Including clinical stage, T stage, N stage, M stage, histological grading, and molecular subtype.

#### 2.3.3 Treatment information

The collected information included surgery, chemotherapy, radiotherapy, targeted therapy, immunotherapy, and endocrine therapy.

#### 2.3.4 Sleep quality

The PSQI scale is a 19-item self-reported instrument developed to assess the subtype and frequency of sleep disturbance, and the overall sleep quality over a 1-month interval (24). It is a comprehensive assessment tool for sleep quality and not a diagnostic tool for clinical Insomnia Disorder (ICSD-3/DSM-5-TR). The total score comprises seven domains: sleep disturbances, sleep latency, sleep efficiency, sleep quality, sleep duration, daytime dysfunction, and sleep medication. Based on the validated PSQI stratification standard for cancer population (25), sleep quality is categorized into three grades using the overall PSQI score: mild sleep disturbance (6–10 points), moderate sleep disturbance (11–15 points), and severe sleep disturbance (16–21 points). This scale demonstrates a diagnostic sensitivity of 89.6% and specificity of 86.5% (26, 27).

#### 2.3.5 Pain

The Visual Analogue Scale (VAS) is an instrument designed to quantify pain intensity using a visual analog scale, widely utilized to evaluate subjective pain perception in breast cancer patients. Patients rate their pain intensity on a 10-cm straight line, where a score of 0 represents no pain and 10 denotes unbearable pain. The Chinese version of VAS exhibits strong internal consistency, as evidenced by a Cronbach’s α coefficient ranging 0.94–0.96, demonstrating good internal consistency (28, 29).

#### 2.3.6 Fatigue

The Revised Piper Fatigue Scale (PFS-R) is used to assess fatigue severity in breast cancer patients. This 22-item scale consists of four dimensions: emotional, behavioral, cognitive, and sensory, with higher scores reflecting more severe fatigue. The Chinese version of PFS-R has good reliability and validity in breast cancer patients, with a Cronbach’s α coefficient and test-retest reliability of 0.98 (30, 31).

#### 2.3.7 Anxiety and depression

The Hospital Anxiety and Depression Scale (HADS) is a commonly employed self-report measure for assessing anxiety and depressive symptoms. It comprises two subscales targeting anxiety and depression. A cut-off score of >7 on each subscale is used to identify the presence of anxiety or depression, with higher scores manifesting greater severity. The Chinese version of HADS exhibits solid internal consistency, with a Cronbach’s α coefficient of ≥0.84 (32, 33).

### 2.4 Statistical analysis

All statistical computations were run in SPSS 24.0 software. Descriptive measures covered constituent proportions, mean ± standard deviation ( $\bar{x}$ ± S), as well as median and quartiles ranges [M(Q25, Q75)]. Shapiro-Wilk test was used to verify the normality of continuous variables. For between-group comparisons (two groups), either the independent-samples t-test or Mann-Whitney U test was applied; for multi-group comparisons, analysis of variance (ANOVA) or Kruskal-Wallis H test was used instead and the Bonferroni correction was adopted for multiple comparison adjustment with the corrected test level set as α’=0.05/k (k is the number of comparisons in each group). Pearson correlation coefficient was used to assess linear associations between normally distributed continuous variables. Variables that showed significant differences in univariate assessments were entered into a multivariate binary unconditional logistic regression model. Box-Tidwell method was used to test the logit linearity of continuous predictors, and variance inflation factor (VIF) was used to test multicollinearity. ROC curve was drawn to evaluate the predictive efficiency of the model (AUC). One-by-one variable elimination method was used to verify the stability of the model results.

## 3 Results

**Figure 1:**
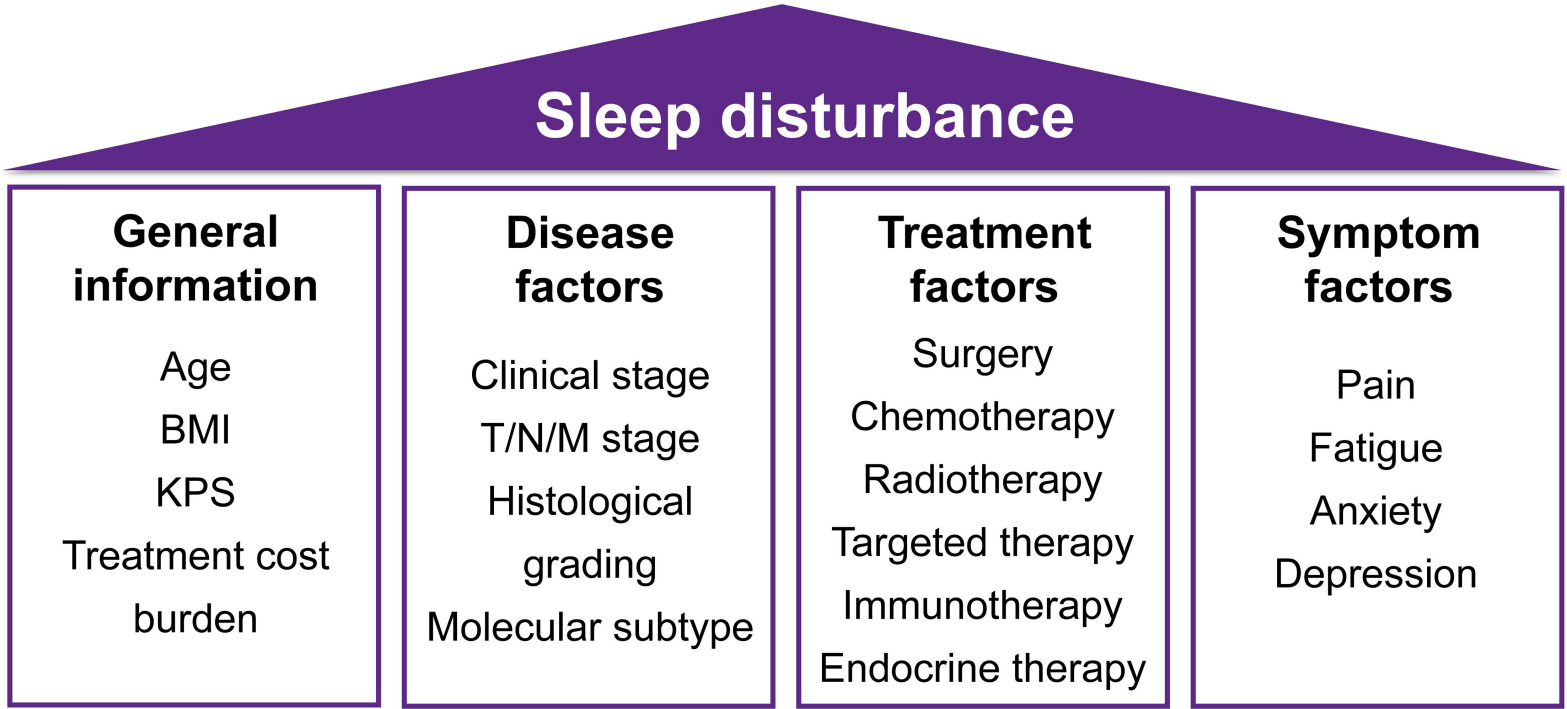
Factors analyzed in this study.

**Figure 2:**
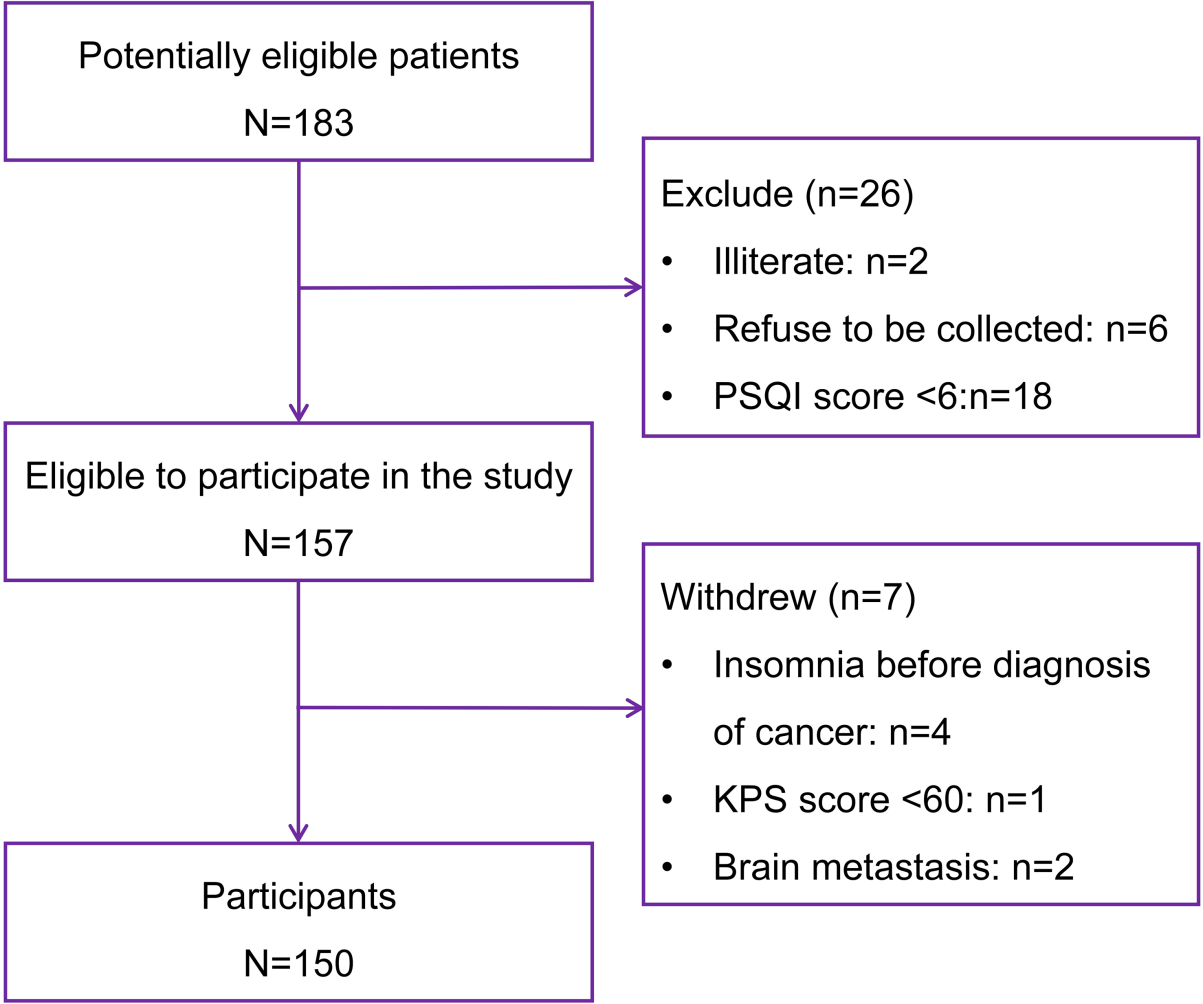
Recruitment process of participants.

Among 183 screened participants, 150 breast cancer patients were ultimately enrolled. Using a crude estimation method (requiring 5–10 participants per independent variable) (25, 34, 35), the required sample size for 20 independent variables (**Figure 1**) ranges from 100 to 200 participants, and the sample size of this study (150 participants) meets this criterion. The recruitment process of participants is illustrated in **Figure 2**. Basic information of the enrolled participants are summarized in **Table 1**.

**Table 1:** Basic information of enrolled participants (n = 150).

Variables	Number	Percent	Variables	Number	Percent
Age			Molecular subtype		
≤40	26	17.33%	Luminal A	42	28.00%
41-60	95	63.33%	Luminal B	60	40.00%
>60	29	19.33%	HER2-positive	21	14.00%
BMI			Triple-negative	27	18.00%
<18.5	3	2.00%	Surgery		
18.5≤ BMI <24	74	49.33%	Yes	148	98.67%
24≤ BMI <28	63	42.00%	No	2	1.33%
≥28	10	6.67%	Chemotherapy		
KPS score			Yes	120	80.00%
90	114	76.00%	No	30	20.00%
80	17	11.33%	Radiotherapy		
70	7	4.67%	Yes	79	52.67%
60	12	8.00%	No	71	47.33%
Treatment Cost Burden			Targeted therapy		
Mild	28	18.67%	Yes	56	37.33%
Moderate	90	60.00%	No	94	62.67%
Severe	32	21.33%	Immunotherapy		
Clinical stage			Yes	2	1.33%
I	83	55.33%	No	148	98.67%
II	43	28.67%	Endocrine therapy		
III	21	14.00%	AI	35	23.33%
IV	3	2.00%	SERM	20	13.33%
T stage			OFS+AI/SERM	29	19.33%
T1	71	47.33%	None	66	44.00%
T2	55	36.67%	PSQI score		
T3	19	12.67%	6-10	47	31.33%
T4	5	3.33%	11-15	74	49.33%
N stage			16-21	29	19.33%
N0	98	65.33%	VAS Score		
N1	31	20.67%	≤4	129	86.00%
N2	9	6.00%	>4	21	14.00%
N3	12	8.00%	PFS-R Score		
M stage			≤4	97	64.67%
M0	147	98.00%	>4	53	35.33%
M1	3	2.00%	HADS anxiety score		
Histological grading			≤7	105	70.00%
I	10	6.67%	>7	45	30.00%
II	66	44.00%	HADS depression score		
III	74	49.33%	≤7	111	74.00%
			>7	39	26.00%

### 3.1 Characteristics of sleep disturbance

**Figure 3:**
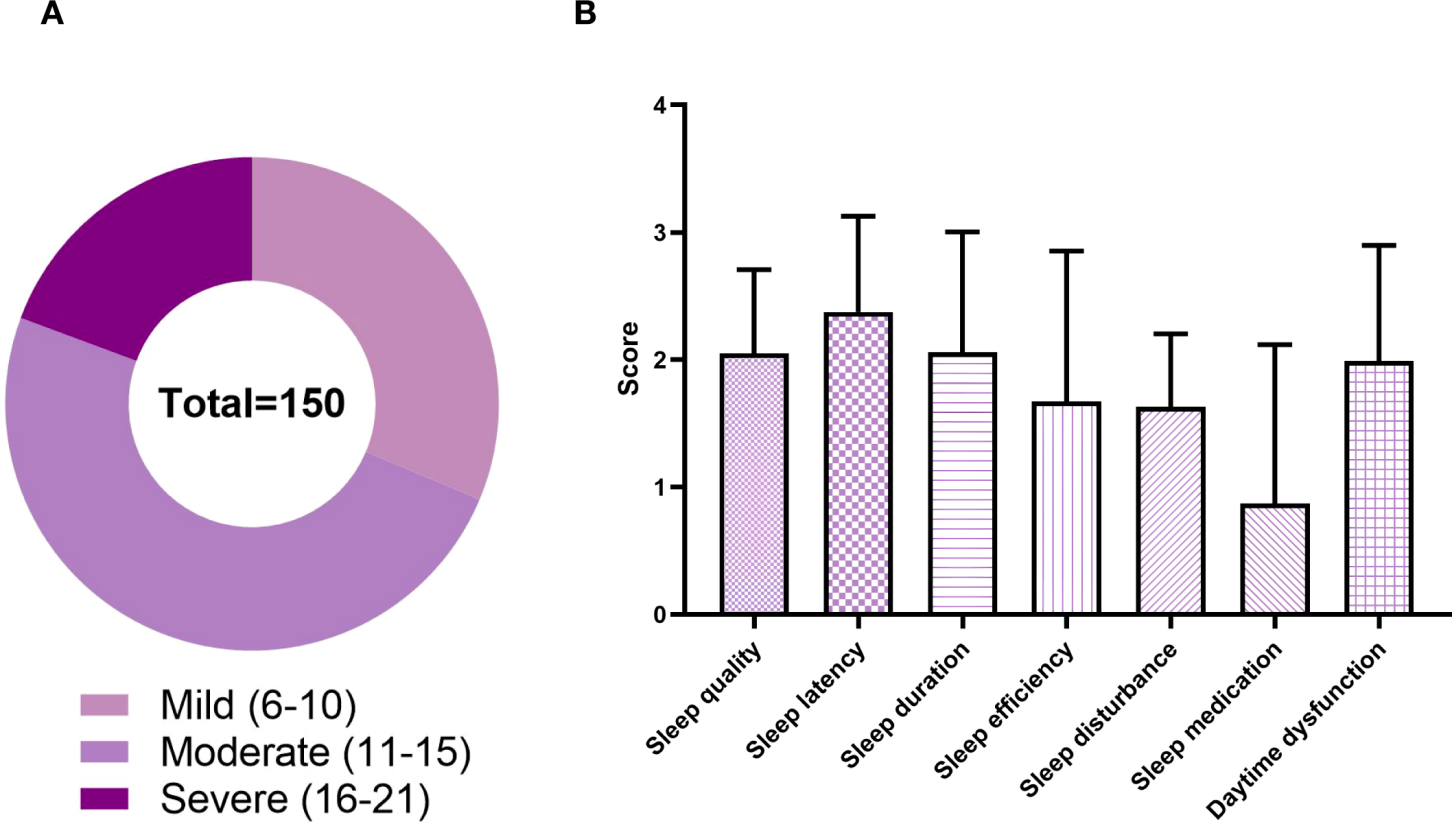
Characteristics of sleep disturbance in breast cancer patients **(A)** Severity: Sleep quality score based on total PSQI. **(B)** Subtypes: Scores of each PSQI component.

The participants had a mean total PSQI score of 12.50 ± 3.64. As many as 68.67% of breast cancer patients exhibited moderate or severe sleep disturbance (i.e., total PSQI score > 10), as shown in **Figure 3A** and **Table 1**. The scores of each PSQI component ranked in descending order were as follows: sleep latency (2.39 ± 0.73) > sleep duration (2.04 ± 1.00) > sleep quality (2.02 ± 0.69) > daytime dysfunction (2.01 ± 0.93) > sleep efficiency (1.61 ± 1.19) > sleep disturbances (1.59 ± 0.57) > sleep medication (0.84 ± 1.25) (**Figure 3B**).

### 3.2 Univariate analysis

#### 3.2.1 General information

Kruskal-Wallis H tests were utilized to compare PSQI scores across different groups categorized by age, Body Mass Index (BMI), KPS score, and treatment cost burden. The results showed H values of 3.208, 0.867, 4.029, and 2.073, respectively, with all corresponding *p*-values > 0.05 (**Table 2**).

**Table 2:** Associations between sleep disturbance and general/disease/treatment information.

Variables	Subgroups	n	PSQI score	H/Z/t	*P*
Age	≤40	26	11.00 (10.00, 13.00)^a^	3.208^c^	0.201
	41-60	95	13.00 (9.50, 15.00)^a^		
	>60	29	12.00 (12.00, 16.00)^a^		
BMI	<18.5	3	14.00 (12.00, 15.00)^a^	0.867^c^	0.834
	18.5≤ BMI <24	74	12.00 (10.00, 15.00)^a^		
	24≤ BMI <28	63	13.00 (9.50, 15.00)^a^		
	≥28	10	12.50 (7.75, 14.75)^a^		
KPS score	90	114	12.00 (10.00, 15.00)^a^	2.346^c^	0.504
	80	17	13.00 (9.00, 15.00)^a^		
	70	7	13.00 (11.50, 14.50)^a^		
	60	12	14.00 (12.75, 15.25)^a^		
Treatment cost burden	Mild	28	13.00 (11.75, 15.00)^a^	2.073^c^	0.355
	Moderate	90	12.00 (10.00, 15.00)^a^		
	Severe	32	12.50 (10.00, 15.25)^a^		
Clinical stage	I	83	13.00 (10.50, 15.00)^a^	5.235^c^	0.155
	II	43	11.00 (9.00, 15.00)^a^		
	III	21	12.00 (10.00, 15.00)^a^		
	IV	3	9.00 (8.00, 11.50)^a^		
T stage	T1	71	13.00 (11.00, 15.00)^a^	6.219^c^	0.101
	T2	55	13.00 (9.50, 15.00)^a^		
	T3	19	12.00 (9.50, 13.00)^a^		
	T4	5	10.00 (10.00, 11.00)^a^		
N stage	N0	98	12.50 (10.00, 15.00)^a^	2.690^c^	0.442
	N1	31	13.00 (10.00, 14.50)^a^		
	N2	9	10.00 (9.00, 12.00)^a^		
	N3	12	13.00 (10.00, 15.00)^a^		
M stage	M0	147	12.00 (10.00, 15.00)^a^	-1.220^d^	0.223
	M1	3	9.00 (8.00, 11.50)^a^		
Histological grading	I	10	12.50 (11.25, 15.00)^a^	1.462^c^	0.481
	II	66	13.00 (10.00, 15.00)^a^		
	III	74	12.00 (10.00, 15.00)^a^		
Molecular subtype	Luminal A	42	13.00 (10.00, 15.00)^a^	1.453^c^	0.693
	Luminal B	60	12.00 (9.75, 15.00)^a^		
	HER2-positive	21	12.00 (10.00, 15.00)^a^		
	Triple-negative	27	12.00 (11.00, 15.00)^a^		
Surgery	Yes	148	12.00 (10.00, 15.00)^a^	-0.214^d^	0.831
	No	2	12.00 (12.00, 12.00)^a^		
Chemotherapy	Yes	120	12.50 (10.00, 15.00)^a^	-0.580^d^	0.562
	No	30	12.00 (9.25, 15.00)^a^		
Radiotherapy	Yes	79	12.45 ± 3.56^b^	-0.180^e^	0.858
	No	71	12.56 ± 3.76^b^		
Targeted therapy	Yes	56	12.00 (10.00, 15.00)^a^	-0.460^d^	0.645
	No	94	12.00 (10.00, 15.00)^a^		
Immunotherapy	Yes	2	13.00 (11.50, 14.50)^a^	-0.247^d^	0.805
	No	148	12.00 (10.00, 15.00)^a^		
Endocrine therapy	AI	35	14.00 (12.00, 16.00)^a^	13.812^c^	0.003^*^
	SERM	20	11.50 (8.75, 13.00)^a^		
	OFS+AI/SERM	29	14.00 (10.75, 15.00)^a^		
	None	66	11.50 (8.00, 15.00)^a^		

^a^M (Q25,Q75), ^b^ $\bar{x}\pm S$, ^c^H value, ^d^Z value, ^e^t value, ^*^p < 0.05.

#### 3.2.2 Disease information

Kruskal-Wallis H tests were conducted to compare PSQI scores across groups stratified by clinical stage, T stage, N stage, histological grade, and molecular subtype. Test outcomes returned H values of 5.235, 6.219, 2.690, 1.462, and 1.453 in sequence, with all corresponding *p*-values > 0.05. A Mann-Whitney U test was applied to assess PSQI scores between M stage groups, yielding a Z value of -1.220 and a *p*-value > 0.05 (**Table 2**).

#### 3.2.3 Treatment information

Mann-Whitney U tests were utilized to evaluate PSQI scores across groups stratified by surgery, chemotherapy, targeted therapy, and immunotherapy. The results showed Z values of -0.214, -0.580, -0.460, and -0.247, respectively, with all corresponding *p*-values > 0.05. An independent samples t-test was used to assess PSQI scores between radiation therapy groups, yielding a t-value of -0.180 and a *p*-value > 0.05 (**Table 2**).

A Kruskal-Wallis H test was performed to compare PSQI scores across endocrine therapy groups, which revealed an H value of 13.812 and a *p*-value of 0.003 (**Table 2**).

#### 3.2.4 Symptom information

**Figure 4:**
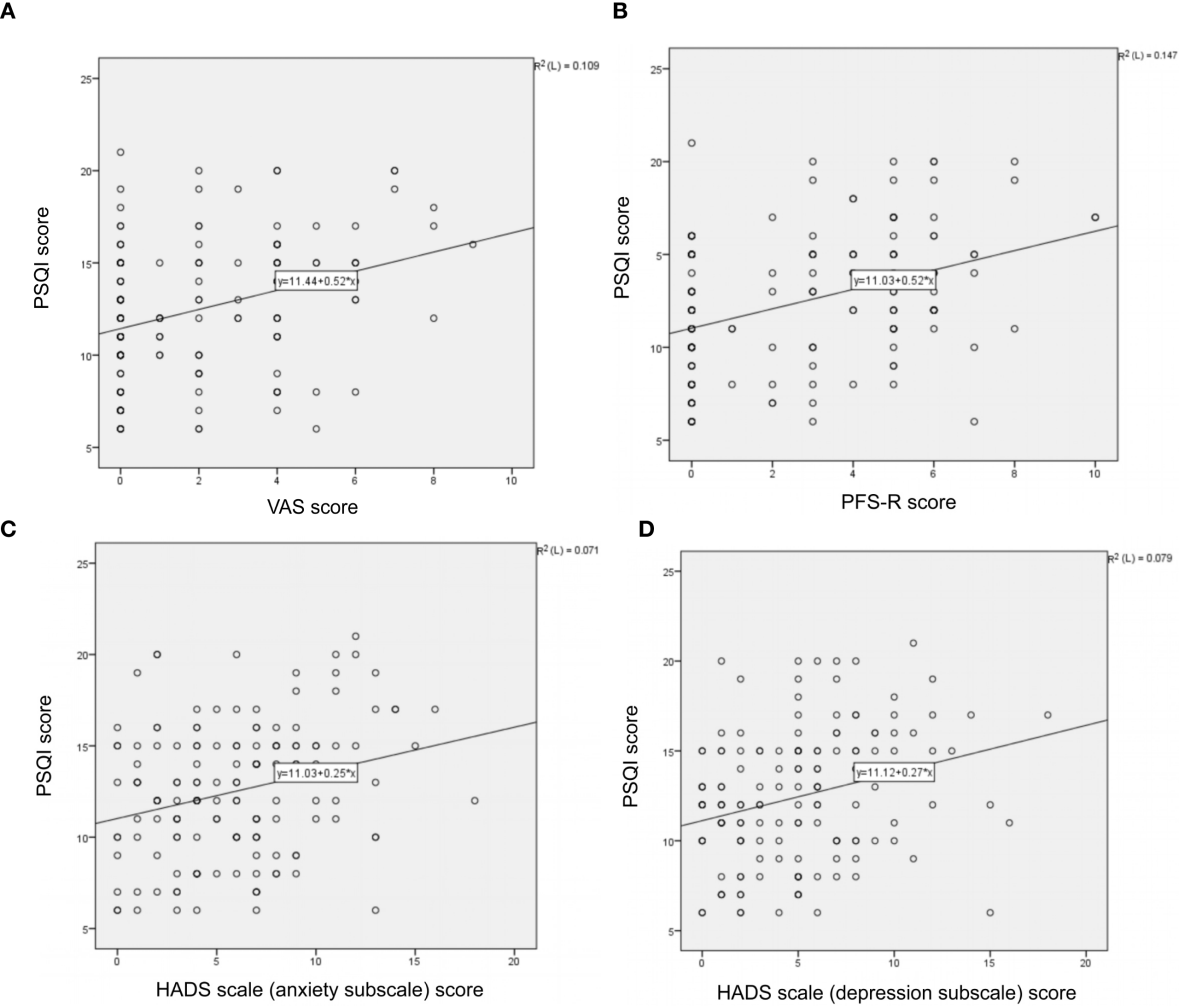
Associations between sleep disturbance and pain **(A)**, fatigue **(B)**, anxiety **(C)**, and depression **(D)**.

Pearson correlation analysis between total PSQI score and the VAS score, PFS-R score, HADS anxiety subscale score, and HADS depression subscale score in breast cancer patients were 0.331, 0.383, 0.266, and 0.282, respectively, with the *p*-values of 0.000, 0.000, 0.001, and 0.000 (**Figure 4**).

### 3.3 Multivariate analysis

Using the forward stepwise selection approach, we built a multivariate binary unconditional logistic regression model. The PSQI score was dichotomized: coded as 0 for PSQI < 16 points (mild/moderate sleep disturbance) and 1 for PSQI ≥ 16 points (severe sleep disturbance). This cutoff was selected because standard PSQI criteria define 16–21 points as severe insomnia requiring targeted intervention, and our study focused on this high-burden subgroup with unmet clinical needs, while mild-to-moderate insomnia is managed with basic sleep hygiene guidance (35). The aforementioned relevant variables with *p* < 0.05 (endocrine therapy, pain, fatigue, anxiety, depression) were incorporated into the model as independent variables.

The results of the Box-Tidwell test showed that the *p*-values of all interaction terms were>0.05, indicating that all continuous variables met the logit linear assumption and were suitable for inclusion in the logistic regression model. The VIF values for pain, fatigue, anxiety, and depression were all below 3, indicating that multicollinearity was not a concern in the model.

**Figure 5:**
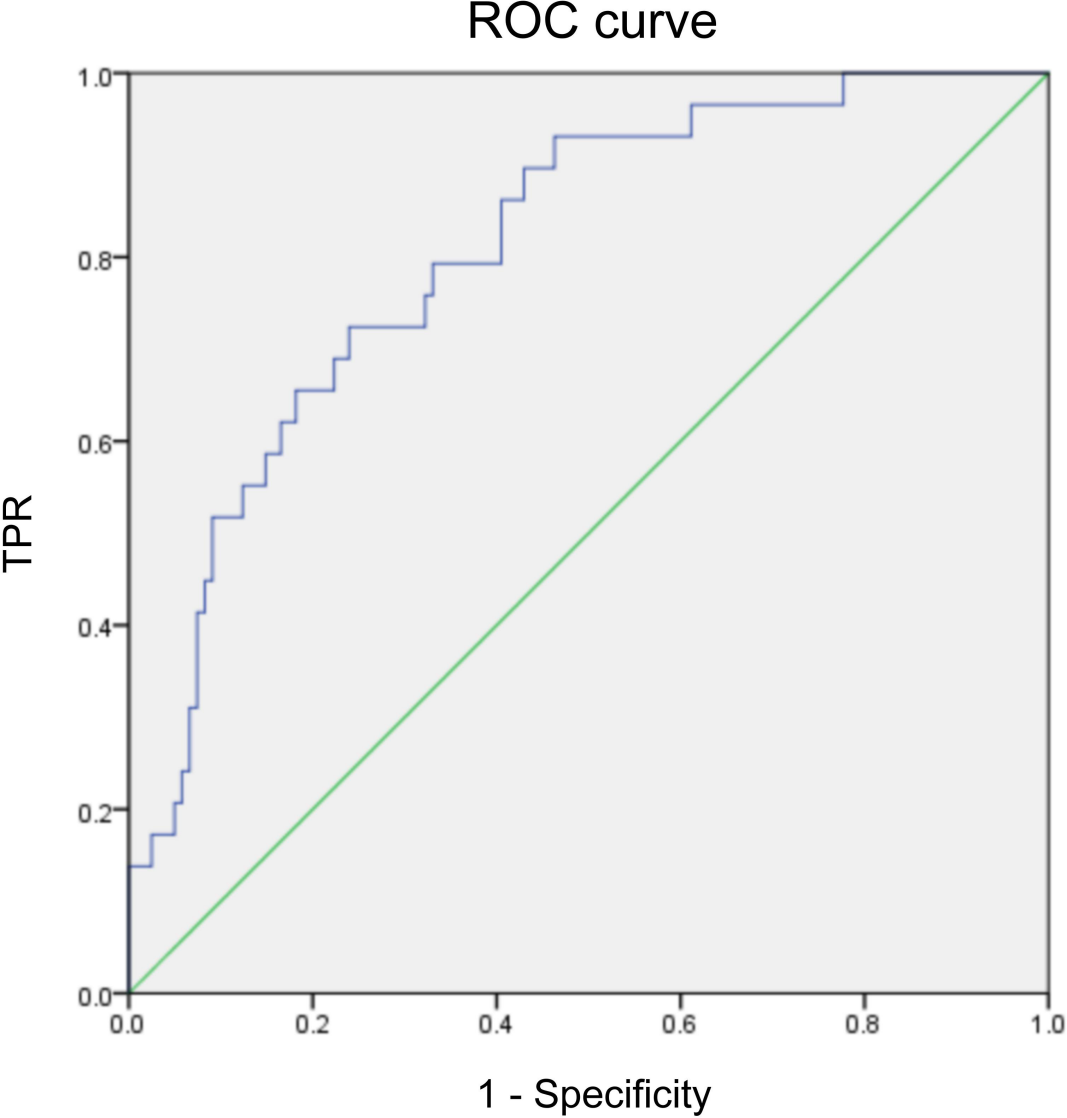
ROC curve.

Regression analysis revealed that pain, fatigue, and depression were significant associated factors for the deterioration of sleep quality. Pain (OR = 1.237, 95% CI: 1.018–1.501, *p* = 0.032), fatigue (OR = 1.250, 95% CI: 1.019–1.535, *p* = 0.033), and depression (OR = 1.237, 95% CI: 1.030–1.487, *p* = 0.023) linked to a higher likelihood of severe sleep disturbance. Specifically, for each 1-point increase in the VAS score, PFS-R score, and HADS depression subscale score, the risk of aggravated sleep disturbance in breast cancer patients increased by 23.7%, 25.0%, and 23.7%, respectively (**Table 3**). The ROC curve of the model showed AUC = 0.808 (95%CI: 0.725-0.890, *p*<0.001), indicating good predictive efficiency (**Figure 5**). Sensitivity analysis (one-by-one variable elimination) showed that the OR values of pain, fatigue and depression had no significant changes, and the model results were stable.

**Table 3:** Multivariate analysis of sleep disturbance.

Factor	B	SE	Wald χ^2^	*P*	OR	OR 95% CI	
Endocrine therapy-None	·	·	2.368	0.500	·	·	·
Endocrine therapy-AI	0.037	0.562	0.004	0.947	1.038	0.345	3.121
Endocrine therapy-SERM	-1.549	1.126	1.89	0.169	0.213	0.023	1.933
Endocrine therapy-OFS+AI/SERM	-0.456	0.653	0.488	0.485	0.634	0.176	2.280
Pain	0.212	0.099	4.597	0.032^*^	1.237	1.018	1.501
Fatigue	0.223	0.105	4.564	0.033^*^	1.250	1.019	1.535
Anxiety	-0.103	0.096	1.140	0.286	0.902	0.747	1.090
Depression	0.213	0.094	5.156	0.023^*^	1.237	1.030	1.487

“·” indicates that compared with AI, SERM, and OFS + AI/SERM, no endocrine therapy was used as the reference category.

^*^p < 0.05.

## 4 Discussion

This study examined the characteristics and influencing factors of sleep disturbance in breast cancer patients. In addition to sleep quality, data on patients’ general information, disease information, treatment information, and symptoms information were also collected. Findings from this study offer insights into the improvement of sleep disturbance in the breast cancer population, provide support for optimizing the comprehensive management of breast cancer, and are of great significance for enhancing the overall quality of life.

### 4.1 Characteristics of sleep disturbance in breast cancer patients

The results of this study indicate that breast cancer patients suffer from severe sleep disturbance, with difficulty falling asleep as the primary manifestation. The mean PSQI score of patients in this study was 12.50, which was significantly higher than that in the general population (10.12) (36), lung cancer patients (10.33) (37), colorectal cancer patients (10.50) (38), endometrial cancer patients (11.11) (39), and gastric cancer patients (11.96) (25). These findings suggest that sleep disturbance is more severe in breast cancer patients relative to the general population and those with other cancer types. Therefore, it is critical to develop targeted interventions to improve their sleep quality. Further research revealed that difficulty falling asleep is the primary feature of sleep disturbance in breast cancer patients, indicating that priority should be given to drugs with rapid onset and short half-lives (such as melatonin receptor agonists and non-benzodiazepine sedative-hypnotic drugs), or non-pharmacological interventions including Cognitive Behavioral Therapy for Insomnia (CBT-I) (40), acupuncture (41), and relaxation training (e.g., Tai Chi, massage, and yoga). Long-acting sedative drugs (such as diazepam and clonazepam) should be avoided (42, 43).

### 4.2 Influencing factors of sleep disturbance in breast cancer patients

#### 4.2.1 Cancer treatment

Univariate analysis in this research reveals that receiving endocrine therapy is a notable factor affecting sleep disturbance in breast cancer patients, though it correlates with neither surgery, chemotherapy, radiotherapy, targeted therapy, or immunotherapy. Ferreira et al. surveyed 4,262 early-stage breast cancer patients and found that endocrine therapy significantly exacerbated pain, sleep disturbance, and side effects of systemic therapy, whereas no such exacerbation of symptoms was observed in patients receiving chemotherapy (44). From the perspective of the mechanism of action, endocrine therapy curbs breast cancer cell growth by lowering bodily estrogen levels or blocking estrogen’s biological activity (45), thereby controlling breast cancer recurrence and prolonging patients’ survival. Existing studies have confirmed that there is a direct neurobiological link between circulating estradiol and sleep; estradiol can maintain the normal operation of circadian rhythms by regulating circadian rhythm-related neural pathways (such as signal transduction in the hypothalamic suprachiasmatic nucleus) (46). Therefore, endocrine therapy may induce estrogen level disorders, interfere with its regulatory effect on circadian rhythms, disrupt the balance of the sleep-wake cycle, and thus become an important cause of inducing or exacerbating sleep disturbance (47, 48).

Further investigation in this work revealed that among breast cancer patients receiving endocrine therapy, the sleep quality of those treated with ovarian function suppression (OFS) or aromatase inhibitor (AI) was significantly lower than that of those treated with SERM. This aligns with prior research findings. Results from a 12-month observational study found that relative to the SERM cohort, the OFS + SERM group saw more significant declines in sleep health over the treatment period (49). In a follow-up study of 1,052 patients with early-stage breast cancer, Li and colleagues noted that patients receiving AI treatment had a significant exacerbation of sleep disturbance starting from the 12th month, whereas no such exacerbation was observed in the SERM group from baseline to the 36th month of treatment (50). This discrepancy likely stems from differing mechanisms: OFS significantly reduces estrogen levels in the body by continuously inhibiting pituitary function and lowering follicle-stimulating hormone (FSH) and luteinizing hormone (LH); AI depletes estrogen levels by inhibiting aromatase activity and blocking the conversion of androgens to estrogens; in contrast, SERM exerts a mixed agonist-antagonist effect by competing with estrogen for binding to estrogen receptors, and its impact on systemic estrogen levels is significantly weaker than that of the previous two (51). This underscores the importance of strengthening the management of sleep disturbance in patients receiving OFS and AI treatment.

However, multivariate analysis in this study showed that endocrine therapy was not an independent risk factor for sleep disturbance, and its association with sleep disturbance may be mediated by the significant factors (pain, fatigue and depression). This indicates that endocrine therapy may induce pain, fatigue and other symptoms by altering hormone levels, and then indirectly aggravate sleep disturbance. This study did not conduct a formal mediating effect analysis, which is a research gap and needs to be verified by subsequent studies with larger samples and more detailed indicators.

#### 4.2.2 Concomitant symptoms

Our findings indicate that sleep disturbance among breast cancer patients was significantly associated with pain, fatigue, depression, and anxiety, among which the first three were key associated factors for sleep disturbance. Each 1-point rise in pain, depression, and fatigue scale scores linked to a 23.7%, 23.7%, and 25.0% higher likelihood of severe sleep disturbance in this group, respectively. Boldyrev et al. surveyed 505 postoperative breast cancer patients and found a strong link between sleep disturbance and pain (52). Dahl et al. further identified arm/shoulder pain as a key predictor of sleep disturbances in this patient group (53). In a study on the correlation between sleep disturbance and fatigue, Dirksen and Epstein found that breast cancer patients with sleep disturbance who received cognitive behavioral therapy (CBT) also achieved significant improvement in fatigue (54). Aggeli et al. reported in a cross-sectional survey of 170 breast cancer patients that sleep disturbance showed moderate positive correlations with both anxiety and depression (55). Desai et al. similarly identified anxiety and depression as significant sleep disturbance risk factors for breast cancer patients on AI therapy (56).

However, it is important to acknowledge the potential bidirectional relationships between sleep disturbance and the concomitant symptoms identified in this study. While our cross-sectional design precludes definitive causal inferences, existing evidence suggests that the associations between sleep disturbance and depression may be reciprocal. For instance, a cross-sectional study showed that higher PSQI scores were significantly associated with higher depression scores (57). Depression itself may exacerbate sleep disturbances through dysregulation of the hypothalamic-pituitary-adrenal (HPA) axis and alterations in circadian rhythm (58). Similarly, the relationship between sleep disturbance and fatigue appears bidirectional: poor sleep quality perpetuates cancer-related fatigue through immunological and neuroendocrine mechanisms, while fatigue may disrupt normal sleep-wake cycles and reduce physical activity, further compromising sleep quality (59). Pain and sleep disturbance also exhibit mutual reinforcement, where pain interferes with sleep continuity and sleep deprivation lowers pain thresholds (60). These bidirectional dynamics suggest that sleep disturbance and its associated symptoms may form a self-perpetuating cycle, underscoring the need for integrated interventions targeting multiple symptoms simultaneously rather than isolated treatment approaches.

Since sleep disturbance, pain, fatigue, and negative emotions often occur as an interconnected symptom cluster among breast cancer patients, scholars have proposed that these symptoms may share underlying pathogenic mechanisms and have defined them collectively as the psychoneurological symptom cluster (61–63). Reports indicate that the incidence of the psychoneurological symptom cluster in breast cancer patients is as high as 42%-45% (19). Studies show that pro-inflammatory cytokines (such as IL-6, TNF-α, and CRP), overactivation of the HPA axis, and serotonin (5-HT) system alterations may drive the emergence of this cluster (19). These findings suggest that clinical management should simultaneously address patients’ fatigue, pain, and emotional issues, and assessment frameworks based on the psychoneurological symptom cluster may serve as effective tools to support holistic clinical care for breast cancer patients.

Notably, breast cancer-related lymphedema represents another important complication that may interact with the symptom cluster identified in this study. Lymphedema, occurring in approximately 20% of breast cancer survivors, has been shown to significantly impair sleep quality through multiple mechanisms (16). Physical discomfort from limb swelling, pain, and restricted mobility can directly disrupt sleep continuity, while the psychological burden of body image disturbance and anxiety related to lymphedema may further exacerbate sleep-onset difficulties (16). Interestingly, complete decongestive therapy (CDT) for lymphedema has demonstrated efficacy in improving both fatigue and sleep quality, suggesting that effective lymphedema management may indirectly benefit sleep disturbance through symptom cluster modulation (64). Although our study did not specifically assess lymphedema status, these findings highlight the importance of screening for lymphedema in breast cancer patients with sleep disturbance, as targeted lymphedema interventions may represent a valuable adjunctive strategy for improving sleep outcomes. Future research should examine the specific contribution of lymphedema to the psychoneurological symptom cluster in breast cancer patients.

### 4.3 Limitations

First, this work was conducted at a single institution, introducing potential selection bias. Second, adopting a cross-sectional design, the associations between sleep disturbance and the aforementioned factors are merely exploratory conclusions, and the potential correlations require validation through further large-sample prospective studies. Third, this study did not retain patients’ blood samples for the detection of inflammatory factors and hormone levels, thus failing to analyze from the perspective of biological indicators. Fourth, small sample groups (immunotherapy n=2, M1 stage n=3) were included in the analysis, and the results may have bias, which cannot reflect the characteristics of sleep disturbance in this population. Fifth, this study used PSQI to assess sleep quality, which is a comprehensive assessment tool rather than a diagnostic instrument for clinical Insomnia Disorder. Since all participants had PSQI ≥6, we only analyzed the severity and related factors of sleep disturbance in this population, and could not estimate the prevalence of insomnia in the overall breast cancer population. Thus, our findings cannot be extrapolated to clinical insomnia diagnosis or epidemiological inference. Sixth, the study did not collect specific data on patients’ occupation, menopausal status, endocrine therapy duration, detailed comorbidities, time since diagnosis, lymphedema status, and the specific usage details of sleep medication, and cannot analyze the influence of these factors on sleep disturbance.

## 5 Conclusion

Breast cancer patients suffer from severe sleep disturbance, primarily characterized by difficulty falling asleep. Univariate analysis showed that their sleep disturbance was associated with receiving OFS/AI endocrine therapy, pain, fatigue, anxiety, and depression, among which pain, fatigue, and depression are independent key associated factors for severe sleep disturbance. Findings from this work can provide a reference for the early detection and prevention of sleep disturbance in breast cancer patients, and simultaneously lay a foundation for formulating effective intervention measures and improving the cancer management system.

## Data Availability

The original contributions presented in the study are included in the article/supplementary material. Further inquiries can be directed to the corresponding author.
